# Hodge decomposition of vector fields in Cartesian grids

**DOI:** 10.1145/3680528.3687602

**Published:** 2024-12-03

**Authors:** ZHE SU, YIYING TONG, GUOWEI WEI

**Affiliations:** Michigan State University, United States of America; Michigan State University, United States of America; Michigan State University, United States of America

**Keywords:** vector field decomposition, Cartesian grids, boundary conditions, discrete exterior calculus, cohomology

## Abstract

While explicit representations of shapes such as triangular and tetrahedral meshes are often used for boundary surfaces and 3D volumes bounded by closed surfaces, implicit representations of planar regions and volumetric regions defined by level-set functions have also found widespread applications in geometric modeling and simulations. However, an important computational tool, the *L*^2^-orthogonal Hodge decomposition for scalar and vector fields defined on implicit representations under commonly used Dirichlet/Neumann boundary conditions with proper correspondence to the topology presents additional challenges. For instance, the projection to the interior or boundary of the domain is not as straightforward as in the mesh-based frameworks. Thus, we introduce a comprehensive 5-component Hodge decomposition that unifies normal and tangential components in the Cartesian representation. Numerical experiments on various objects, including singlecell RNA velocity, validate the effectiveness of our approach, confirming the expected rigorous *L*^2^-orthogonality and the accurate cohomology.

## 1 Introduction

The Hodge decomposition of vector fields into components with specific properties has a broad range of applications. For instance, it plays a crucial role in computational fluid dynamics [Yang et al. 2021; Yin et al. 2023], flow processing and visualization [Sawhney and Crane 2020], geometric modeling [Wang and Chern 2021], spectral data analysis [Keros and Subr 2023], and machine learning [Su et al. 2024]. As a prototypical example, vector fields on a compact domain in the 2D or 3D Euclidean space can be orthogonally decomposed into divergence-free and curl-free components, as in the Helmholtz-Hodge decomposition. The general form of the decomposition based on Hodge’s foundational work [1989] applies to differential forms (covariant antisymmetric tensor fields), which can be regarded as a generalization of scalar and vector fields. It decomposes the space of differential *k*-forms into a direct sum of *L*^2^-orthogonal subspaces.

On closed manifolds, the classical Hodge decomposition [Hodge 1989] involves Laplacians, which are second-order linear operators with finite-dimensional kernels called harmonic spaces. The dimensions of these kernels correspond to the topology of the underlying manifolds, leading to implementations through linear systems with finite rank deficiency. However, on manifolds with boundary, the situation becomes subtle. The spaces of harmonic fields are infinite-dimensional, resulting in linear systems with substantial rank deficiency. To mitigate this issue, specific boundary conditions, such as the normal (Dirichlet) and tangential (Neumann) boundary conditions, have been introduced [Shonkwiler 2009]. Under these conditions, kernels are again finite-dimensional, with correspondences to the topology of the manifold and its boundary.

The domains for vector field processing through finite element type approaches in geometric modeling and computer graphics [Poelke and Polthier 2016; Tong et al. 2003] are frequently represented by simplicial meshes, e.g., the unstructured surface or volume meshes resulting from either Lagrangian or Eulerian formulations of fluid simulation. On the other hand, structured meshes such as Cartesian grids are advantageous in other types of discretization, including finite-volume, finite-difference, and pseudo-spectral methods [Fedkiw et al. 2001; Liu et al. 2015; Stam 2023], as well as convolutional neural networks on voxelized data. However, for the complete 5-component Hodge decomposition of vector fields on compact domains, the existing methods [Poelke 2017; Razafindrazaka et al. 2019; Zhao et al. 2019] rely exclusively on simplicial mesh discretization frameworks, where the underlying manifolds are modeled either by triangular or tetrahedral meshes. To facilitate the direct application of the 5-component Hodge decomposition on a compact domain defined within a regular Cartesian grid, we propose linear systems directly assembled based on a given level-set function, without resorting to tessellation and data conversion.


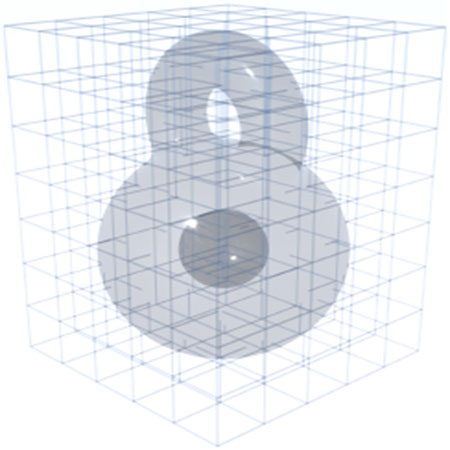


### 1.1 Related work

The Hodge decomposition for smooth manifolds with boundary has been thoroughly discussed in [Schwarz 2006] with up to 4 components. A refinement of the decomposition to five terms is presented in [Shonkwiler 2013], which offers insights on further splitting the cohomology groups (which correspond to the kernels of Laplacians) into portions derived from the interior and boundary of the manifold. An elementary exposition of this 5-component Hodge decomposition for compact domains in ${\mathbb{R}}^{3}$ in terms of vector and scalar fields can be found in [Cantarella et al. 2002].

In the discrete case, the Helmholtz-Hodge decomposition (HHD) has been implemented in various methods. In [Polthier and Preuß 2000], a global variational approach was used for HHD of piecewise constant vector fields on triangulated surfaces, by *L*^2^-projection to the spaces of curl-free and divergence-free components. It was then extended to the 3D case on tetrahedral meshes [Polthier and Preuß 2003; Tong et al. 2003], and to the 2D case on regular grids [Guo et al. 2005]. A meshless algorithm of HHD was introduced in [Petronetto et al. 2009] for point vectors in ${\mathbb{R}}^{2}$. See [Bhatia et al. 2012; De Goes et al. 2016] for a comprehensive discussion on the theory and practice of HHD for 2D and 3D vector fields.

The complete 5-component discrete Hodge decomposition was subsequently introduced for piecewise constant vector fields, for surface triangle meshes [Poelke and Polthier 2016], and for tetrahedral meshes [Poelke 2017]. The framework aligns well with the smooth case, accurately capturing the topological structures. The decomposition can be done more efficiently when using Whitney bases representations for differential forms, such as Nedelec elements on edges, and Raviart-Thomas elements on faces [Razafindrazaka et al. 2019; Zhao et al. 2019].

### 1.2 Contributions

We aim to fill the gap for the 5-component Hodge decomposition on Cartesian grids with 2D/3D domains defined by level set functions. Unlike existing methods, we do not require an explicit tessellation of the domain into simplicial meshes, eliminating the reliance on high-quality meshing tools and streamlining the decomposition. Moreover, we maintain the pairwise *L*^2^-orthogonality among the components while preserving the consistency with the topology.

## 2 Hodge decomposition in the smooth case

Before describing the discretization, we briefly review the continuous theory. Let M be an m-dimensional smooth, orientable, compact manifold with boundary. In particular, when M is a compact domain in ${\mathbb{R}}^{m}$, m=2or3 with its metric induced by Euclidean metric, any vector field w on M can be decomposed as w=∇a+∇×b, where a is a scalar field and b is a vector field. The curl-free component ∇a and the divergence-free component ∇×b are $L^{2}$-orthogonal with each other under certain boundary conditions detailed later, and the decomposition is unique with certain assumptions on the topology of M.

The generalization of this crucial mathematical tool in scientific computing to higher dimensions and generic manifolds is often through differential *k*-forms, which can be interpreted as an entity that can be integrated on *k*-submanifolds, or equivalently an anti-symmetric covariant tensor field of rank *k* defined on *M*. Specifically, in ${\mathbb{R}}^{2}$, a 0-form or a 2-form can be identified with a scalar field and a 1-form can be identified with a vector field, while in ${\mathbb{R}}^{3}$, a 0-form or 3-form can be identified with a scalar field and a 1-form or 2-form can be regarded as a vector field.

The space of all differential *k*-forms on M is denoted as $\Omega^{k}$. The differential d, also called exterior derivative, is the unique ℝ-linear mapping from k-forms to (k+1)-forms satisfying the Leibniz rule with respect to the wedge product ∧ (antisymmetric tensor product) and the nilpotent property dd=0. This operator generalizes and unifies several differential operators in vector analysis, including gradient ∇, curl ∇×, and divergence ∇⋅, which correspond to d applied to 0-, 1-, and 2-forms in ${\mathbb{R}}^{3}$ respectively. The identity dd=0, corresponds to the vector field analysis identities ∇⋅∇×=0 and ∇×∇=0. See [Desbrun et al. 2006] for a comprehensive comparison.

A differential form $\omega \in \Omega^{k}$ is *closed* if dω=0 and *exact* if there is a (k−1)-form $\zeta \in \Omega^{k-1}$ such that ω=dζ. Every exact form is closed due to dd=0. The integral of an exact form dω over an oriented k+1-submanifold S⊂M with boundary ∂S can be reduced to a boundary integral of ω, according to Stokes’ theorem, a generalization of the Newton-Leibniz rule,

(2.1) $$\int_{S}d\omega =\int_{\partial S}\omega .$$

Given a Riemannian metric g on M, any k-form may be identified with a unique (n−k)-form through the Hodge star $⋆\;:\;\Omega^{k}\to \Omega^{n-k}$, the unique linear operator satisfying

(2.2) $$\omega \wedge ⋆\eta ={\langle \omega ,\eta \rangle }_{g}\;\mu_{g},$$

where $\omega ,\eta \in \Omega^{k},\langle \cdot ,\cdot \rangle_{g}$ denotes the pointwise inner product induced by g on $\Omega^{k}$, and $\mu_{g}$ is the volume form induced by g. By integrating Eq. (2.2), we obtain the Hodge $L^{2}$-inner product on the space of k-forms $\Omega^{k}$

(2.3) $$(\omega ,\eta )=\int_{M}\omega \wedge ⋆\eta .$$

With the differential d and the Hodge star ⋆, the codifferential operator $\delta \;:\;\Omega^{k}\to \Omega^{k-1}$ can be defined as

(2.4) $$\delta ={\left(-1\right)}^{m(k-1)+1}⋆d⋆.$$

The codifferential δ in ${\mathbb{R}}^{3}$ also corresponds to −∇,∇×, and −∇⋅ when applied to 2-, 1-, 0-forms respectively. It is also nilpotent, δδ=0. A differential form $\omega \in \Omega^{k}$ is *coclosed* if δω=0 and *coexact* if there is a (k+1)-form $\eta \in \Omega^{k+1}$ such that ω=δη. The Hodge Laplacian is then defined as $\Delta =d\delta +\delta d\;:\;\Omega^{k}\to \Omega^{k}$. In ${\mathbb{R}}^{3}$, they correspond to scalar and vector Laplacians up to a sign change for k=0, 3 and k=1, 2 respectively. A k-form ω is *harmonic* if Δω=0.

### 2.1 Hodge decomposition for closed manifolds

The classical Hodge decomposition theorem states that the space of differential *k*-forms can be decomposed orthogonally as

(2.5) $${\text{Ω}}^{k}=d{\text{Ω}}^{k-1}⊕\delta {\text{Ω}}^{k+1}⊕{ℋ}_{\text{Δ}}^{k}(M),$$

where the space ${ℋ}_{\Delta }^{k}(M)$ is the finite-dimensional kernel of Δ. The orthogonality can be established from

(2.6) $$(d\alpha ,\beta )-(\alpha ,\delta \beta )=\int_{\partial M}\alpha \wedge ⋆\beta ,$$

for any $\alpha \in \Omega^{k-1}$ and $\beta \in \Omega^{k}$. When ∂M=∅, the above indicates any exact (coexact) form is orthogonal to coclosed (closed resp.) forms. The dimension of ${ℋ}_{\Delta }^{k}(M)$ is determined through the Hodge isomorphism with the k-th de Rham cohomology, $H_{dR}^{k}(M)=$ $ker\;d^{k}/Im\;d^{k-1}$, the quotient space of closed k-forms modulo exact k-forms. It follows from the de Rham theorem and Poincaré duality that the de Rham cohomology group $H_{dR}^{k}(M)$ is isomorphic to the (m−k)-singular homology group, whose dimension is given by the (m−k)-th Betti number $\beta_{m-k}$, where $\beta_{0},\beta_{1},\beta_{2}$ provide the numbers of connected components, tunnels, and cavities respectively. Therefore, $dim\;{ℋ}_{\Delta }^{k}(M)=\beta_{m-k}$.

### 2.2 Hodge decomposition for manifolds with boundary

#### 2.2.1 Orthogonality.

With a nonempty boundary, the orthogonality is not guaranteed unless boundary conditions are imposed to the r.h.s of Eq. 2.6. Two such subspaces can be defined: $\Omega_{n}^{k}$ and $\Omega_{t}^{k}$ for the homogeneous normal and tangential boundary conditions,

(2.7) $${\text{Ω}}_{n}^{k}=\left\{\omega \in {\text{Ω}}^{k}|\omega {|}_{\partial M}=0\right\},\;{\text{Ω}}_{t}^{k}=\left\{\omega \in {\text{Ω}}^{k}|⋆\omega {|}_{\partial M}=0\right\}.$$

For instance, velocity fields represented by tangential 1-forms satisfy the no-transfer boundary conditions, whereas its vorticity fields represented by normal 1-forms have streamlines orthogonal to the boundary. It follows from the definitions that $⋆\;:\;\Omega_{n}^{k}\to \Omega_{t}^{m-k}$ provides an isomorphism between the two. In addition, d preserves the normal boundary condition, whereas δ preserves the tangential boundary condition. The Hodge-Morrey decomposition [Morrey 1956], which decomposes a k-form into an exact normal form, a coexact tangential form, and the rest, is thus orthogonal,

(2.8) $${\text{Ω}}^{k}=d{\text{Ω}}_{n}^{k-1}⊕\delta {\text{Ω}}_{t}^{k+1}⊕{ℋ}^{k},$$

where ${ℋ}^{k}=ker\;d\cap ker\;\delta$ is the space of closed and coclosed fields. However, ${ℋ}^{k}\subset {ℋ}_{\Delta }^{k}(M)$ is infinite-dimensional [Schwarz 2006].

#### 2.2.2 Complete decomposition.

The solution is to enforce boundary conditions on the harmonic space, and decompose the space ${ℋ}^{k}$ further into three terms [Friedrichs 1955]

(2.9) $${ℋ}^{k}=\left({ℋ}_{n}^{k}+{ℋ}_{t}^{k}\right)⊕\left(d{\text{Ω}}^{k-1}\cap {\delta \text{Ω}}^{k+1}\right),$$

where, the subspace ${ℋ}_{n}^{k}={ℋ}^{k}\cap \Omega_{n}^{k}(M)$ (or ${ℋ}_{t}^{k}={ℋ}^{k}\cap \Omega_{t}^{k}(M)$, resp.) under the normal (or tangential) boundary conditions, is finite-dimensional. The spaces ${ℋ}_{n}^{k}$ and ${ℋ}_{t}^{k}$, in general, are not orthogonal. However, they are orthogonal on compact domains in Euclidean spaces [Shonkwiler 2009]. Thus, on such domains, a 5-component Hodge decomposition is available,

(2.10) $${\text{Ω}}^{k}=d{\text{Ω}}_{n}^{k-1}⊕\delta {\text{Ω}}_{t}^{k+1}⊕{ℋ}_{n}^{k}⊕{ℋ}_{t}^{k}⊕\left(d{\text{Ω}}^{k-1}\cap \delta {\text{Ω}}^{k+1}\right).$$

Given $\omega \in \Omega^{k}$, by the 5-component Hodge decomposition (2.10), there is a unique orthogonal 5 component decomposition (2.3):

(2.11) $$\omega =d\alpha_{n}+\delta \beta_{t}+h_{n}+h_{t}+\eta ,$$

where $\alpha_{n}\in \Omega_{n}^{k-1},\beta_{t}\in \Omega_{t}^{k+1},h_{n}\in {ℋ}_{n}^{k},h_{t}\in {ℋ}_{t}^{k}$ and $\eta \;\in \;d\Omega^{k-1}\cap \delta \Omega^{k+1}$. The first two terms can be computed by first solving for the potentials $\alpha_{n}\in \Omega_{n}^{k-1}$ and $\beta_{t}\in \Omega_{t}^{k+1}$, and then applying the differential d to $\alpha_{n}$ and codifferential δ to $\beta_{t}$. Note that these potentials are not uniquely determined as $d\left(\alpha_{n}+d\xi \right)=d\left(\alpha_{n}\right)$ and $\delta \left(\beta_{t}+\delta \zeta \right)=\delta \beta_{t}$ for all $\xi \in \Omega_{n}^{k-2}$ and $\zeta \in \Omega_{t}^{k+2}$. To ensure the uniqueness of these potentials, we impose gauge conditions $\delta \alpha_{n}=0$ and dβ=0. We then obtain

(2.12) $$\delta \omega =(\delta d+d\delta )\alpha_{n}=\text{Δ}\alpha_{n},\;d\omega =(\delta d+d\delta )\beta_{t}=\text{Δ}\beta_{t}.$$

By resolving the rank deficiencies of Laplacians on $\Omega_{n}^{k-1}$ and $\Omega_{t}^{k+1}$ (see Sec 3.3.1), the potential $\alpha_{n}$ can then be solved by considering the first equation in (2.12) with boundary conditions ${\left.\alpha_{n}\right|}_{\partial M}=0$ and ${\left.\delta \alpha_{n}\right|}_{\partial M}=0$, while $\beta_{t}$ can be solved by using the second equation with boundary conditions ${\left.⋆\beta_{t}\right|}_{\partial M}=0$ and ${\left.⋆d\beta_{t}\right|}_{\partial M}=0$.

Since ${ℋ}_{n}^{k}$ and ${ℋ}_{t}^{k}$ are finite-dimensional, $h_{n}$ and $h_{t}$ can be calculated by projecting the form ω onto the spaces ${ℋ}_{n}^{k}$ and ${ℋ}_{t}^{k}$ in any orthonormal bases. The last term η can then be calculated by subtracting these four terms from ω.

The 5-component decomposition can also be performed in two steps, by a 3-component decomposition,

(2.13) $${\text{Ω}}^{k}=d{\text{Ω}}^{k-1}⊕\delta {\text{Ω}}_{t}^{k+1}⊕{ℋ}_{t}^{k}\;=d{\text{Ω}}_{n}^{k-1}⊕\delta {\text{Ω}}^{k+1}⊕{ℋ}_{n}^{k},$$

followed by further decomposing Imd or Imδ,

(2.14) $$\text{Im}\;d=d{\text{Ω}}_{n}^{k-1}⊕{ℋ}_{n}^{k}⊕\left(d{\text{Ω}}^{k-1}\cap \delta {\text{Ω}}^{k+1}\right),$$

(2.15) $$\text{Im}\;\delta =\delta {\text{Ω}}_{t}^{k+1}⊕{ℋ}_{t}^{k}⊕\left(d{\text{Ω}}^{k-1}\cap \delta {\text{Ω}}^{k+1}\right).$$

#### 2.2.3 Dimensionality of tangential/normal kernels.

Each tangential (or normal) harmonic field corresponds to a unique equivalence class in the absolute (or respectively relative) de Rham cohomology, i.e., ${ℋ}_{t}^{k}≅H_{dR}^{k}(M)$ and ${ℋ}_{n}^{k}≅H_{dR}^{k}(M,\partial M)$ [Friedrichs 1955]. It follows that, for compact manifolds in ${\mathbb{R}}^{m}$, the dimensions of these subspaces are given by the Betti numbers $dim\;{ℋ}_{n}^{k}=\beta_{m-k}$ and $dim\;{ℋ}_{t}^{k}=\beta_{k}$, fully determined by the topology.

## 3 Discretization of the Hodge decomposition

The generalization of discrete exterior calculus (DEC) [Desbrun et al. 2006; Wang et al. 2023] from simplicial meshes to regular grids is straightforward (see inset for an example of the boundary operator on a grid face). Our focus is on 2D/3D domains bounded by a level set curve/surface embedded within such grids. In fact, the interpretation of the discrete forms and discrete differential based on boundary operator is particularly intuitive for regular grids. Up to a constant scaling factor, discrete 0- and 3-forms correspond to scalar fields sampled on grid points and on cell centers; discrete 1- and 2-forms correspond to vector fields its components sampled on *X*, *Y*, *Z* grid edges, and those on *YZ*, *ZX*, *XY* grid faces. Discrete differential operator corresponds to grad/curl/div when applied to 0/1/2-forms, e.g., for an *XY* grid face representing *Z*-component of ∇×w, the sum of w over the four edges corresponds to the circulation around the face, or equivalently, $\frac{\partial w_{y}}{\partial x}-\frac{\partial w_{x}}{\partial y}$ times the area of the face.


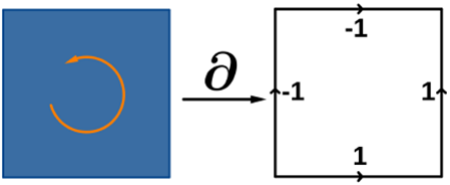


### 3.1 Discretization on grids

#### 3.1.1 Discrete forms.

Let $I_{m}$ be a rectangular m-dim regular Cartesian grid with k-cells oriented according to their alignment with the coordinate axes. With uniform grid spacing l along each axis direction, each k-cell of the grid is a k-dimensional hypercube. With the grid $I_{m}$ treated as a cell complex tessellating a rectangular domain in ${\mathbb{R}}^{m}$, a continuous k-form ω can be discretized by its integral value $W^{i}=\int_{\sigma_{i}}\omega$ over each k-cell $\sigma_{i}$ [Desbrun et al. 2006]. Let $c=\sum_{i}a_{i}\sigma_{i}$ be a k-chain, given as a formal linear combination of k-cells representing a k-dimensional subdomain. Then the discretization, known as the de Rham map, linearly maps a k-form to a cochain, which is a linear functional that maps the k-chain c to the integral of ω over c, i.e, $\int_{c}\omega =\sum_{i}W^{i}a_{i}$.

#### 3.1.2 Discrete differential operator.

The discrete differential operator acting on discrete k-forms is represented by a sparse matrix $D_{k}^{I}$. This matrix encodes the signed incidence between (k+1)-cells and k-cells, as in the simplicial case, i.e., $D_{k}^{I}=\partial_{k+1}^{T}$, the transpose of the cell boundary operator $\partial_{k+1}$ on (k+1)-cells. This is the direct consequence of Stokes’ theorem $\int_{\sigma }d\omega =\int_{\partial \sigma }\omega$. It follows that $D_{k+1}^{I}D_{k}^{I}=0$, since the boundary of a boundary of any cell is a 0 chain (i.e., ∂∂=0).

#### 3.1.3 Discrete Hodge star.

Treating the centers of m-cells as grid points, we construct a *dual* grid that is staggered with the *primal* grid $I_{m}$. A one-to-one correspondence between discrete k-forms on the primal grid and (m−k)-form on the dual grid can be established by the diagonal discrete Hodge star ${⋆}_{k}$, induced by the continuous Hodge star through local averaging

(3.1) $$\frac{1}{\left|\sigma_{k}\right|}\int_{\sigma_{k}}\omega \approx \frac{1}{\left|⋆\sigma_{k}\right|}\int_{⋆\sigma_{k}}⋆\omega ,$$

where $⋆\sigma_{k}$ is the dual (m−k)-cell formed by the dual grid points associated with the m-cells incident to the k-cell $\sigma_{k}$. Equivalent to assuming a one-point quadrature for the integration involved in discretizing primal and dual forms, this correspondence leads to a diagonal matrix $S_{k}^{I}$ with diagonal entries given by the ratio between the volumes of the dual (m−k)-cells and primal k-cells, $l^{m-k}/l^{k}=l^{m-2k}$. The associated discrete Hodge $L^{2}$ inner product (2.3) of two discrete k-forms $V_{k}$ and $W_{k}$ on the grid $I_{m}$ is then

(3.2) $${\left(V_{k},W_{k}\right)}^{I}=V_{k}^{T}S_{k}^{I}W_{k}.$$

#### 3.1.4 Discrete Laplacian.

The discrete codifferential operator δ can be assembled from the discrete differential and Hodge star operators as ${\left(S_{k-1}^{I}\right)}^{-1}{\left(D_{k-1}^{I}\right)}^{T}S_{k}^{I}$. With the differential and codifferential in Δ=dδ+δd replaced by their discrete counterparts, the resulting matrix is nonsymmetric. Therefore, the discrete Laplacian is defined as the counterpart of ⋆Δ,

(3.3) $$L_{k}^{I}={\left(D_{k}^{I}\right)}^{T}S_{k+1}^{I}D_{k}^{I}+S_{k}^{I}D_{k-1}^{I}{\left(S_{k-1}^{I}\right)}^{-1}{\left(D_{k-1}^{I}\right)}^{T}S_{k}^{I},$$

where the operator is considered null for k<0 or k>m.

### 3.2 Discrete differential forms and operators on *M*

For simplicial or polygonal meshes, identifying boundary elements is straightforward, allowing easy implementation of projection matrices to the interior or boundary of the domain *M*. However, when *M* is defined by the volume enclosed within a level set surface, enforcing boundary conditions through projection matrices becomes challenging. Instead of tessellating the boundary cells to form new unstructured meshes, e.g., through Marching Cubes, we modify the Hodge star operators. This approach maintains consistent data structures, accommodating evolving level sets and eliminating the need for remeshing.

#### 3.2.1 Compact supports.

While we do not cut the boundary cells, we still need to restrict the computation to the relevant cells through the inclusion or exclusion of the entire *k*-cells, similar to voxelization. However, boundary (primal or dual) *k*-cells typically intersect the boundary rather than being completely contained within it. As we aim to construct Laplacian operators with rank deficiencies corresponding to the topology of *M*, we design one rule for each of the two types of boundary conditions. For normal boundary conditions, we include every cell with at least one vertex inside *M*, while for tangential boundary conditions, we select every cell with at least one vertex of its dual cells inside *M*. The set of cells for the former is called the *normal support* (inset left) and the set of cells for the latter is called the *tangential support* (inset right). Note that, the normal and tangential supports are typically distinct, and, unlike the mesh case, neither is necessarily a superset of the other.


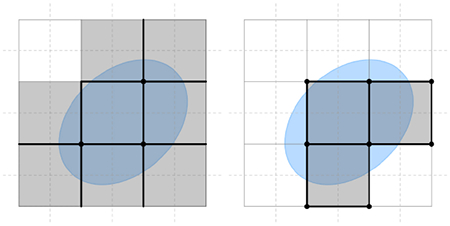


The 0-1 projection matrices $P_{k,n}$, and $P_{k,t}$ map k-chains to the normal and tangential support respectively. They can be derived from the identity matrix by eliminating the rows corresponding to k-cells outside the support. The relevant differential operators are

(3.4) $$D_{k,n}=P_{k+1,n}D_{k}^{I}P_{k,n}^{T},\;D_{k,t}=P_{k+1,t}D_{k}^{I}P_{k,t}^{T}.$$

The nilpotent property $D_{k+1,n}D_{k,n}=0$ and $D_{k+1,t}D_{k,t}=0$ remains for both, due to $D_{k+1}^{I}D_{K}^{I}=0$ and the following observations,

(3.5) $$P_{k+1,n}^{T}P_{k+1,n}D_{k}^{I}P_{k,n}^{T}=D_{k}^{I}P_{k,n}^{T},\;P_{k+1,t}D_{k}^{I}P_{k,t}^{T}P_{k,t}=P_{k+1,t}D_{k}^{I}.$$

#### 3.2.2 Modified Hodge stars and Laplacians.

Our discretization extends the formulations in [Batty et al. 2007; Liu et al. 2015; Ng et al. 2009; Ribando-Gros et al. 2022] to accommodate the 5-component Hodge decomposition. This complete decomposition necessitates the simultaneous use of both tangential fields and normal fields. One key observation is that $d\Omega_{n}$ and $\text{δ}\Omega_{t}$ are essential to the decompositions (Eqs. (2.13) and (2.10)). Thus, while the forms on normal and tangential supports may be “voxelized,” their differentials and codifferentials, respectively, need to effectively approximate elements of $d\Omega_{n}$ and $d\Omega_{t}$.

To this end, we retain the dual cell volumes while adjusting the primal cell volumes for normal boundary conditions, and do the opposite for tangential boundary conditions: keep the primal cell volumes while modifying the dual cell volumes. For instance, a gradient field $\nabla f_{n}$ of a scalar function $f_{n}$ fixed to 0 on the boundary can be represented by $D_{0,n}F_{n}$ of a normal 0-form $F_{n}$. Its value in a boundary cell will be forced to be orthogonal to the boundary as shown in the inset figure. Equivalently, the codifferential of a 3-form with tangential support also satisfies the tangential condition of the resulting 2-form, which also corresponds to a gradient vector field orthogonal to the boundary.


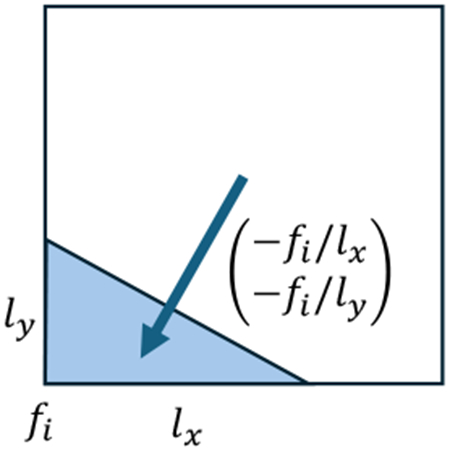


Specifically, for normal (or tangential) boundary conditions, we replace the k-volume of each primal (resp. dual) k-cell. Instead of the full k-volume of $l^{k}$, where l is the grid spacing, we use the k-volume of its intersection with M. This can be computed as the volume of the convex hull formed by the grid points of a k-cell inside M and the intersection points on the grid edges of that k-cell. For the discrete Hodge star matrix, this modified volume is placed in the denominator (or numerator) while the dual (or primal) cell volumes $l^{n-k}$ are left unchanged in the numerator (or denominator). For numerical stability, we perturb the level set function evaluated at primal/dual gridpoints to have an absolute value above $\epsilon =10^{-5}l$, which ensures that fractional k-volumes behave well under double precision. We denote by $S_{k,n}$ and $S_{k,t}$ the resulting sparse Hodge star matrices defined on the normal and tangential supports, respectively.

The discrete $L^{2}$ inner products of the two types of discrete k-forms on the manifold M under these two boundary conditions, namely, the discrete $\Omega_{n}^{k}(M)$ and $\Omega_{t}^{k}(M)$, are thus

(3.6) $${\left(\xi^{k},\zeta^{k}\right)}^{n}={\left(\xi^{k}\right)}^{T}S_{k,n}\zeta^{k},\;{\left(\xi^{k},\zeta^{k}\right)}^{t}={\left(\xi^{k}\right)}^{T}S_{k,t}\zeta^{k}.$$

Finally, we assemble the discrete Hodge Laplacians as follows:

(3.7) $$L_{k,n}=D_{k,n}^{T}S_{k+1,n}D_{k,n}+S_{k,n}D_{k-1,n}S_{k-1,n}^{-1}D_{k-1,n}^{T}S_{k,n}$$

(3.8) $$L_{k,t}=D_{k,t}^{T}S_{k+1,t}D_{k,t}+S_{k,t}D_{k-1,t}S_{k-1,t}^{-1}D_{k-1,t}^{T}S_{k,t}.$$

These two types of discrete Hodge Laplacians are crucial for computing potentials in our implementation of the Hodge decomposition in Eq. (2.8).

### 3.3 Vector field decomposition

In 2D or 3D, vector fields on M can be represented as discrete 1-forms. Following the typical de Rham map $W^{i}=\int_{\sigma_{i}}\omega$, the integral of the vector field along each grid edge is the corresponding primal 1-form (on the normal support). Assume the input vector field is sampled as one vector per grid point in M, and 0 at grid points outside. Then the discrete 1-form on each primal edge in the normal support is the average of the edge direction component of vectors on the inside endpoints of each grid edge, multiplied by the fraction of the primal edge length within the domain. The resulting discrete 1-form is denoted as $W_{n}$ even though ω is not necessarily normal.

To reconstruct the field at a specific point, e.g., in the 3D case, each component of the vector can be evaluated from bilinear interpolation of the average values on the 4 edges along that direction incident to the containing grid cell. This can be seen as the Whitney map on the Cartesian grid, similar to the one that constructs a continuous field from a discrete 1-form on a simplicial mesh.

In 3D, it is possible to use a discrete 2-form instead. While the DoFs for 1-forms and 2-forms can be drastically different in the simplicial mesh case, in our Cartesian representation, it is strictly equivalent to a discrete 1-form on a grid shifted by (l/2,l/2,l/2). Thus, we limit our discussion to the 1-form representation.

**Remark 3.1.** More precisely, a vector field v can also be represented as a 2-form on M by averaging the normal components of vectors on inside grid points of each face times the face area inside the domain. The decomposition of the 2-form following (2.8) provides a dual version, where the Laplacians $L_{1,n}$ and $L_{3,t}$ will be used for solving the vector and scalar potentials, respectively. One could use either of these two representations of vector fields for the Hodge decomposition since they are equivalent to one another through the duality between a Cartesian grid and its staggered dual by offsetting each grid point with (l/2,l/2,l/2), so long as M is at least one grid spacing away from the boundary of the grid.

For the tangential representation of the 1-form, we actually follow the discretization of the normal 2-form as described above on the dual grid, except that the average is rescaled by l instead of by the inside part of the face area. This ensures that the tangential 1-form $W_{t}$ samples the vector field at least at one point within the domain, as at least one of the four neighboring dual cell centers will be inside M. Recall that while $W_{n}$ and $W_{t}$ are merely represented in the normal and tangential supports, respectively, neither necessarily satisfy the corresponding boundary conditions.

In the following, we first describe a naive approach for calculating the five components in the Hodge decomposition Eq. (2.11), which converges to orthogonal components only in the continuous limit. Then, we describe a modified computation that achieves discrete $L^{2}$-orthogonality of the 5 subspaces, mirroring the orthogonality found in mesh-based decomposition.

#### 3.3.1 Direct approach.

Similar to the mesh setting, to solve for the scalar potentials $A_{n}$ defined on vertices (i.e., 0-cells) and $B_{t}$ defined on faces (i.e., 2-cells) in the Hodge decomposition Eq. (2.11), we use the discrete equivalents of Eq. (2.12):

(3.9) $$L_{0,n}A_{n}=D_{0,n}^{T}S_{1,n}W_{n},\;L_{2,t}B_{t}=S_{2,t}D_{1,t}W_{t}.$$

The normal Laplacian $L_{0,n}$ is full rank, but the kernel size of $L_{2,t}$ in 3D is $\beta_{2}$, as determined by the topology of M. To address the rank deficiency, we add a small positive value to $\beta_{2}$ selected diagonal entries to $L_{2,t}$. Following the computation of the potentials, the first two terms in Hodge decomposition (2.11) are calculated by applying the discrete differential $D_{0,n}$ to $A_{n}$ and the discrete codifferential $\delta_{2,t}=S_{1,t}^{-1}D_{1,t}^{T}S_{2,t}$ to $B_{t}$.

To compute the normal harmonic component $N_{h}$ and the tangential harmonic component $T_{h}$, we simply project W to the kernels of the discrete Laplacians $L_{1,n}$ and $L_{1,t}$. As with the mesh-based approach, these two Laplacians correctly capture the cohomology of the underlying manifold: the space of discrete normal harmonic 1-fields is isomorphic to the first relative cohomology group, and the space of discrete tangential harmonic 1-fields isomorphic to the first absolute cohomology group, i.e.,

(3.10) $$\text{ker}\;L_{1,n}≅H_{dR}^{1}(M,\partial M),\;\text{ker}\;L_{1,t}≅H_{dR}^{1}(M).$$

It follows that $dim\;ker\;L_{1,n}=\beta_{m-1}$ and $dim\;ker\;L_{1,t}=\beta_{1}$. The $\beta_{m-1}$ (resp. $\beta_{1}$) eigenvectors corresponding to the 0 eigenvalues of $S_{1,n}^{-1}L_{1,n}$ (resp. $S_{1,t}^{-1}L_{1,t}$) form a basis of the normal (resp. tangential) harmonic space ${ℋ}_{n}^{1}$ (resp. $\left.{ℋ}_{t}^{1}\right)$. Let ${ℍ}_{1,n}$ (resp. $\left.{ℍ}_{1,t}\right)$ be the matrix with columns formed by the basis elements of ${ℋ}_{n}^{1}$ (resp. ${ℋ}_{t}^{1}$). The projections are

(3.11) $$N_{h}={ℍ}_{1,n}{ℍ}_{1,n}^{T}S_{1,n}W_{n},\;T_{h}={ℍ}_{1,t}{ℍ}_{1,t}^{T}S_{1,t}W_{t}.$$

To compute the final term E in (2.11), it is important to account for the different dimensions between the discrete normal forms $D_{0,n}A_{n},N_{h}$ and tangential forms $S_{1,t}^{-1}D_{1,t}^{T}S_{2,t}B_{t},\;T_{h}$. This difference in dimensions arrives due to the relation between the normal and tangential support. Consequently, one has to resolve the consistency issue, e.g., by converting all representations to tangential support. To avoid excessive averaging, we construct the linear conversion operator $C_{n\to t}$ as follows: if an edge is in both supports, we simply rescale the normal 1-form value by $l^{2-m}S_{1,n}$, otherwise, the vectors at the incident cell centers are reconstructed from the normal 1-form (i.e., Whitney map), then the tangential 1-form discretization procedure is carried out on that edge. With this conversion operator,

(3.12) $$E=W_{t}-C_{n\to t}D_{0,n}A_{n}-\delta_{2,t}B_{t}-C_{n\to t}N_{h}-T_{h}.$$

However, the resulting 5 components are only $L^{2}$-orthogonal in the continuous limit.

#### 3.3.2 Discrete orthogonal decomposition.

To achieve a discrete L2-orthogonal decomposition, we first have to choose a consistent L2-inner-product and a consistent functional space. Given that the Hodge duality on regular Cartesian grids is straightforwardly established by shifting the grid by half a grid spacing, we use $S_{1,t}$ and the tangential support for the following discussion without loss of generality.

#### 3.3.3 Tangential decomposition.

According to Eq. (2.13), a 3-component discrete orthogonal decomposition containing two of the components we need is

(3.13) $$W_{t}=D_{0,t}A_{t}+\delta_{2,t}B_{t}+T_{h},$$

where $A_{t}$ and $B_{t}$ can be solved from

(3.14) $$L_{0,t}A_{t}=D_{0,t}^{T}S_{1,t}W_{t},\;L_{2,t}B_{t}=S_{2,t}D_{1,t}W_{t},$$

where the rank deficiency of $L_{0,t}$ is $\beta_{0}$, the number of connected components, which can be fixed in the same way as $L_{2,t}$. The third term $T_{h}$ can be obtained either from the projection procedure Eq. (3.5), or by

(3.15) $$T_{h}=W_{t}-D_{0,t}A_{t}-\delta_{2,t}B_{t}.$$

#### 3.3.4 Gradient field decomposition.

The 5-component decomposition can be regarded as the 3-component tangential decomposition followed by the decomposition of the gradient field space into 3 subspaces. Accordingly, we further decompose $D_{0,t}A_{t}$ into three components,

(3.16) $$D_{0,t}A_{t}=D_{0,t}{\tilde{A}}_{n}+{\tilde{N}}_{h}+\tilde{E}=D_{0,t}\left({\tilde{A}}_{n}+{\tilde{A}}_{h}+{\tilde{A}}_{E}\right),$$

where ${\tilde{A}}_{n}$ is a normal 0-form on the tangential support, approximating a function that vanishes at the boundary, similar to $A_{n}$ except not defined on the normal support; ${\tilde{N}}_{h}=D_{0,t}{\tilde{A}}_{h}$ is similar to $N_{h}$ but on tangential support with the potential ${\tilde{A}}_{h}$; and $\tilde{E}=D_{0,t}{\tilde{A}}_{E}$ is similar to E but defined on tangential support with its scalar potential ${\tilde{A}}_{E}$. Note that all these three components have potentials since they are in subspaces of Imd.

To implement this, we build two nested linear subspaces of $Im\;D_{0,t}$. The first subspace, representing the discrete version of $Im\;D_{0,t}\cap {ℋ}^{1}$, is the space onto which an $L^{2}$-projection will produce ${\tilde{A}}_{h}+{\tilde{A}}_{E}$. The second space is a subspace of the first, denoted as $\tilde{H_{1,n}}$, corresponding to an $L^{2}$-projection providing ${\tilde{A}}_{h}$. The final component can then be computed through ${\tilde{A}}_{n}=A_{t}-\left({\tilde{A}}_{h}+{\tilde{A}}_{E}\right)$. All these potentials are unique, up to one constant shift per connected component (kernel of $\left.L_{0,t}\right)$.

As the first linear subspace is the space of harmonic exact 1-forms, we seek to express it through discrete harmonic 0-form potentials, which are determined by its restriction to the boundary. However, in contrast to the mesh-based case, not all boundary grid points are present in the tangential support of 0-forms. Therefore, we establish an extended support for the harmonic potential A, which includes all grid points incident to any grid edge in either tangential or normal support. With the projection $P_{E\to T}$ representing the projection from the extended support to the tangential support, ${\tilde{A}}_{E}+{\tilde{A}}_{h}=P_{E\to T}A$ is the harmonic potential whose differential leads to $\tilde{E}+{\tilde{N}}_{h}$. The linear space is thus defined as $\left\{A\;|\;L_{0,E}A=0\right\}$, where $L_{0,E}$ is the graph Laplacian for 0-forms in the extended support evaluated on all grid points in the normal support. The graph Laplacian is necessary and sufficient here since we are enforcing neither the tangential nor the normal boundary condition for this component.

The projection to the first linear subspace can be obtained from the linear system resulting from the constrained minimization,

(3.17) $$\underset{A}{\min}\;{\left\|D_{0,t}P_{E\to T}A-D_{0,t}A_{t}\right\|}_{t}^{2}+{\text{Λ}}^{T}L_{0,E}A,$$

where $\|W{\|}_{t}=\sqrt{W^{T}S_{1,t}W}$ is the L2-norm for a 1-form W in tangential support, and $\Lambda^{T}$ is the Lagrange multiplier to enforce the constraint $L_{0,E}A=0$. To eliminate rank deficiency in the linear system, A is set to 0 at one vertex per connected component within tangential support.

The second subspace is constructed as a subspace of normal harmonic forms, which is a subspace of the space of exact harmonic forms. As the dimension is low ($\beta_{2}$), it is efficient to construct its basis. We first find, for the i-th basis 1-form $N_{h,i}$ for the harmonic normal 1-form space, the closest 1-form $D_{0,t}{\overset{‾}{A}}_{h,i}$ within the tangential support can be obtained by first solving

(3.18) $$L_{0,t}{\overset{‾}{A}}_{h,i}=D_{0,t}^{T}S_{1,t}C_{n\to t}N_{h,i},$$

We then project $D_{0,t}{\overset{‾}{A}}_{h,i}$ to the first subspace using Eq. 3.17, and perform the Gram-Schmidt procedure to form an orthonormal basis for $\tilde{H_{1,n}}$. The resulting basis 1-forms can be assembled as ${\tilde{ℍ}}_{1,n}$. The projection is thus

(3.19) $${\tilde{N}}_{h}={\tilde{ℍ}}_{1,n}{\tilde{ℍ}}_{1,n}^{T}S_{1,t}D_{0,t}P_{E\to T}A.$$

### 3.4 Kernel dimensions and $L^{2}$-orthogonality

#### 3.4.1 Voxelized cell complex.

In contrast to the Lagrangian case in [Zhao et al. 2019], the discrete version of de Rham’s theorem on the isomorphism between homology and de Rham cohomology is not immediately apparent on the normal/tangential supports of Cartesian grids. However, while the “voxelized” supports do not follow the actual boundary surface, the cohomology (kerD/ImD) still exists as $D_{t}D_{t}=0\left(D_{n}D_{n}=0\right)$ still holds, and it depends only on $D_{t}($ or $\left.D_{n}\right)$ but not on $S_{t}$ (or resp. $S_{n}$). In fact, as the tangential support contains each primal k-cell that has a dual (n−k)-cell with at least one internal dual grid point, it forms a voxelized cell complex mesh. This is due to that any k-cell in the tangential support has each of its (k−1)-faces also in the tangential support, because the dual (n−k+1)-cell has at least one internal dual grid point. Thus, $ker\;D_{k,t}/Im\;D_{k-1,t}$ is isomorphic to $H^{k}(M)$, since the voxelized mesh is homeomorphic to M. The cohomology on the normal support $ker\;D_{k,n}/Im\;D_{k-1,n}$ likewise corresponds to a voxelized dual cell complex, and is thus isomorphic to $H_{n-k}(M)≅H^{k}(M,\partial M)$.

As $S_{t}$ and $S_{n}$ are nonsingular by construction, it then follows that $ker\;L_{k,t}≅H^{k}(M)$ (and $ker\;L_{k,n}≅H^{k}(M,\partial M)$), since for either $L_{k}$, $\ker\;L_{k}$ contains one unique representative from every equivalence class [W+DF] in $\ker\;D_{k}/Im\;D_{k-1}$ of the associated D. For instance, if both W and $W+D_{0,t}F$ belong to $ker\;L_{1,t}$, then $\delta_{1,t}D_{0,t}F=0$; so F belongs to the kernel of $L_{0,t}$ (constant functions on each connected component), thus $D_{0,t}F=0$.

#### 3.4.2 Orthogonality.

The naive approach does not have strict discrete orthogonality among the 5 subspaces, as they are defined on different types of supports with different Hodge stars as inner products. Neither $L^{2}$-inner product provided strict orthogonality among these components, as confirmed in our experiments. In the following, we show why our projection-based approach establishes the $S_{1,t}$-orthogonality in the decomposition on the tangential support.

By mimicking the Eq. (2.6) on tangential representations, we show the orthogonality among $D_{0,t}A,\delta_{2,t}B_{t}$ and $T_{h}$ as follows

(3.20) $${\left(D_{0,t}A\right)}^{T}S_{1,t}\delta_{2,t}B_{t}=A^{T}S_{0,t}\left(\delta_{1,t}\delta_{2,t}\right)B_{t}=0,$$

(3.21) $${\left(D_{0,t}A\right)}^{T}S_{1,t}T_{h}=A^{T}S_{0,t}\left(\delta_{1,t}T_{h}\right)=0,$$

(3.22) $${\left(\delta_{2,t}B\right)}^{T}S_{1,t}T_{h}=B^{T}S_{2,t}\left(D_{1,t}T_{h}\right)=0.$$

The first orthogonality results from the nilpotent property of δ, and the second and third from the closed and coclosed property of $T_{h}$. The orthogonality of the 5 -component decomposition then follows from the fact that we constructed 3 mutually orthogonal subspaces of $Im\;D_{0,t}$.

## 4 Numerical experiments

Our algorithm is implemented in MATLAB and tested on a laptop with 16GB OF memory. The 5 components are pairwise orthogonal, with an $L^{2}$-inner product of 0 up to the precision of the linear solvers used. Among the examples below, the worst case $L^{2}$-inner product (normalized by the product of the norms of the two components involved) is below $10^{-10}$. The runtime is within 5 seconds for 100×100 grids and 30×30×30 grids, and within 40 seconds for 50×50×50 grids. The images were rendered in Blender. To demonstrate the effectiveness, we provide both 2D and 3D examples of Hodge decomposition of vector fields defined on compact domains. These domains are represented by signed distance functions (SDF) on regular Cartesian grids. Denoting by ρ the level set function, the compact domain is

(4.1) M={x∣ρ(x)≤0},

with the boundary given by ∂M={x∣ρ(x)=0}.

**Fig. 1.**
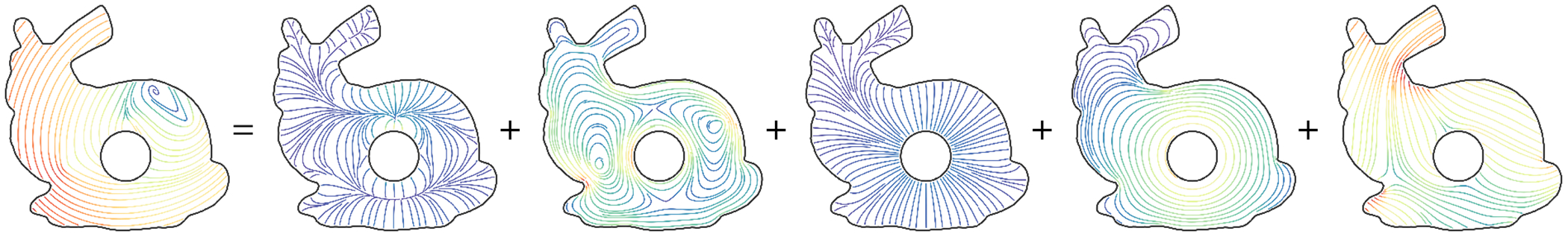
5-component Hodge decomposition. From left to right: the original vector field, the normal gradient field, the tangential curl field, the normal harmonic field, the tangential harmonic field, and the curly gradient field.

In Fig. 1, the 5-component Hodge decomposition is computed in our approach on a 2D bunny-shaped domain with one hole. Both the normal harmonic space ${ℋ}_{n}$ and the tangential harmonic space ${ℋ}_{t}$ are one-dimensional, since $\beta_{1}=1$ due to the annulus topology.

**Fig. 2.**
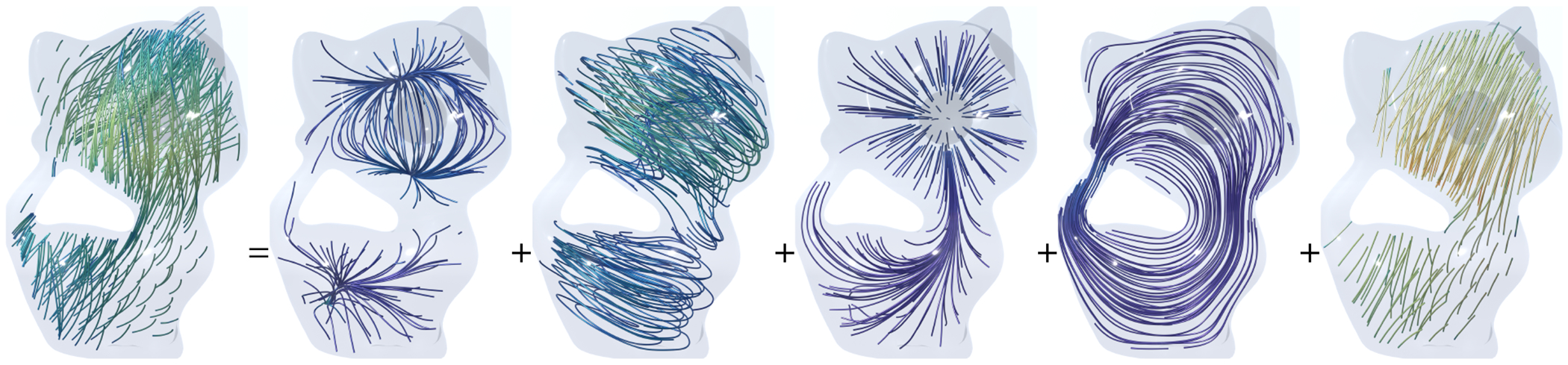
3D Hodge decomposition on kitty model. From left to right: the original vector field, the normal gradient field, the tangential curl field, the normal harmonic field, the tangential harmonic field, and the curly gradient field.

**Fig. 3.**
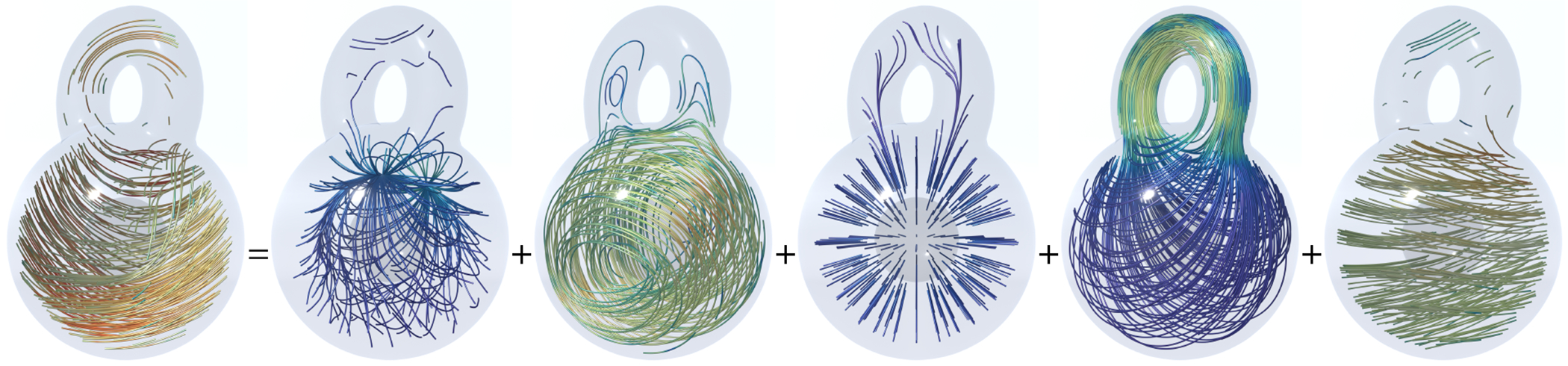
3D Hodge decomposition on kettlebell shape. From left to right: the original vector field, the normal gradient field, the tangential curl field, the normal harmonic field, the tangential harmonic field, and the curly gradient field.

**Fig. 4.**
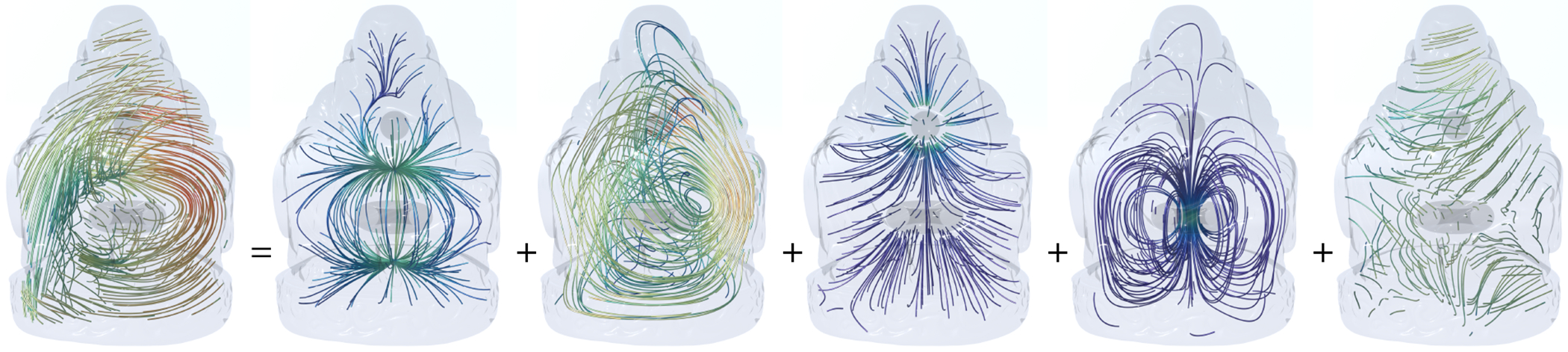
3D Hodge decomposition on Buddha model. To create variations in the topology, a ball and a torus are cut from the inside.

**Fig. 5.**
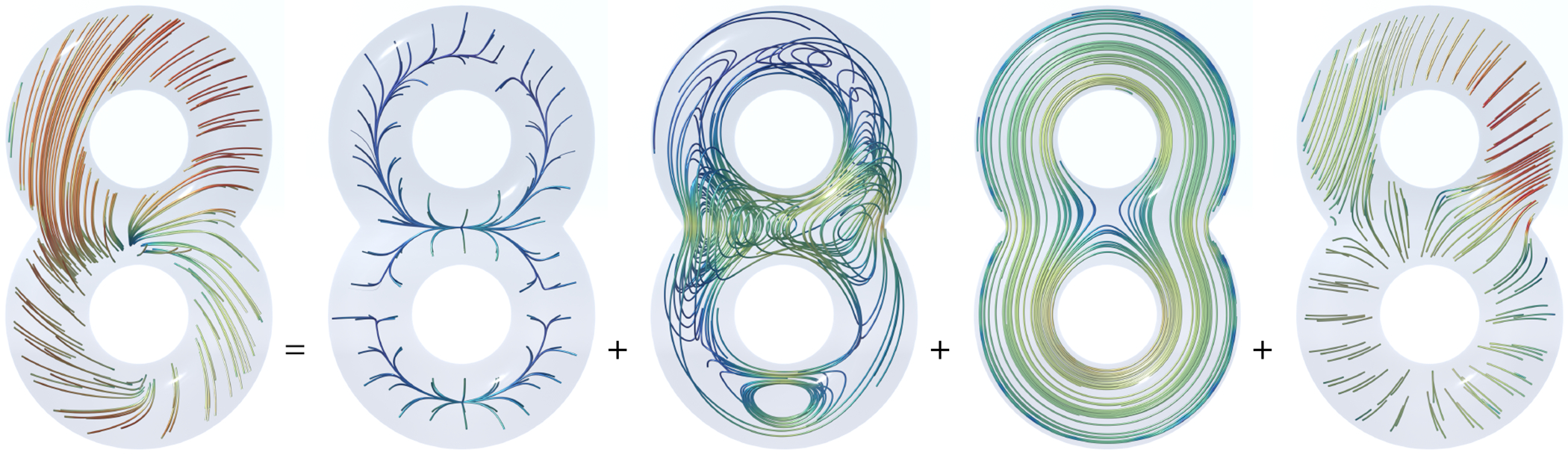
4-component Hodge decomposition on figure 8. From left to right: the original vector field, the normal gradient field, the tangential curl field, the tangential harmonic field, and the curly gradient field. There is no normal harmonic field, as the boundary has a single connected component $\left(\beta_{2}=0\right)$.

**Fig. 6.**
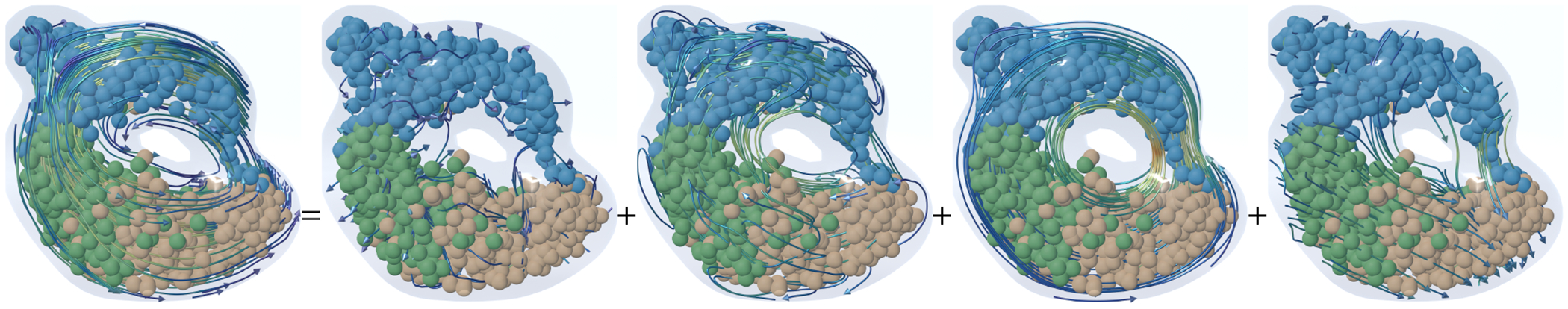
4-component Hodge decomposition on single-cell RNA velocity. There is no normal harmonic field, as the boundary has a single connected component ($\beta_{2}=0$).

In Fig. 2, a vector field on a kitty-shaped domain with a spherical cavity and one handle is decomposed into five components. With $\beta_{1}=1$ and $\beta_{2}=1$, all 5 components can be nonzero. Figs. 3 and 4 show the decomposition on topologically modified kettlebell and buddha models, both with nonzero $\beta_{1}$ and $\beta_{2}$. For the 3D figure-8 model in Fig. 5, $\beta_{1}=2$ but $\beta_{2}=0$, thus the space of normal harmonic fields $h_{t}$ vanishes, leaving only four nonzero components. To demonstrate that the algorithm also applies to abstract manifolds, we show in Fig. 6 an RNA velocity field, which captures the cell dynamic information in the biological processes [Su et al. 2024].

## 5 Conclusion

We have presented a framework to compute the complete 5-component orthogonal decomposition of 2D and 3D vector fields on domains embedded in regular Cartesian grids. We leveraged the correspondence between vector fields and differential forms with certain boundary conditions. The domain boundaries are encoded by isosurfaces of level-set functions. Compared to the methods on simplicial meshes [Razafindrazaka et al. 2019; Zhao et al. 2019], our framework greatly simplifies the data structure and discrete operators by using the vertices, edges, faces, and cells of the grid. This significantly improves the efficiency of our algorithms for potentially evolving level set functions. Our adaptation DEC ensures that the discrete Hodge decomposition preserves the crucial topology structure while conforming to specified boundary conditions. All components can be calculated by solving sparse linear systems with rank efficiency eliminated by accounting for the corresponding spaces of harmonic fields that depend only on the underlying manifold topology. The *L*^2^-orthogonality of the decomposed 5 discrete components is also rigorously guaranteed by the linear algebra formulation. This framework has the potential to benefit various downstream applications due to the ubiquity of vector field analysis on domains in Euclidean spaces.

### Limitations and future work.

Our framework has so far been evaluated only on compact domains in ${\mathbb{R}}^{2}/{\mathbb{R}}^{3}$. Extending it to higher dimensions should be relatively straightforward. However, a limitation lies in the domain representation: it cannot capture sharp features unless the grid resolution is sufficiently fine because the domain is modeled as a region bounded by an isocurve or an isosurface of a level-set function. Periodic domains with obstacles are not implemented either. The geometric details are also constrained by grid resolution. Therefore, one possible future extension is to incorporate adaptive data structures similar to octrees. Moreover, we only considered the Hodge decomposition with each component under just either normal or tangential boundary conditions. Future work could involve generalizing this to mixed boundary conditions as in [Zhao et al. 2019] for the mesh setting. Another interesting variation worth exploring is the implementation of high-order Galerkin-type Hodge stars rather than diagonal ones, and studying their impact on the convergence rate. A possible speedup for the saddle point problem in Eq. (3.17) is through the Schur complement reduction [Benzi et al. 2005] initialized with our direct approach.

## CCS Concepts:

• **Mathematics of computing → Discretization**.
